# Solar-driven aromatic aldehydes: green production from mandelic acid derivatives by a Co(ii)/C_3_N_4_ combined catalyst in aqueous media†

**DOI:** 10.1039/d1ra08256f

**Published:** 2022-02-14

**Authors:** Mi Wu, Hongzhao Wang, Haifang Mao, Chaoyang Wang, Zhenbiao Dong, Ting Tang, Wei Zheng, Lehong Jin, Jibo Liu

**Affiliations:** School of Chemical and Environmental Engineering, Shanghai Institute of Technology 100 Haiquan Road Shanghai 201418 China mhf@sit.edu.cn jiboliu@sit.edu.cn +86-21-60877281; Hangzhou Normal University, College of Medicine 2318 Yuhangtang Road Hangzhou Zhejiang China tangtinghnu@163.com

## Abstract

According to the requirements for sustainable development, reclaiming fine chemicals from wastewater under mild conditions is an extremely significant line of research. A low-cost and high-efficiency polydentate chelate- and polymeric Co(ii)-based complex (Co-L)-loaded C_3_N_4_ photocatalyst (Co-L/C_3_N_4_) was constructed and used to convert aromatic mandelic acids in wastewater at room temperature. The BET specific surface area increased from 28 m^2^ g^−1^ to 68 m^2^ g^−1^, indicating its excellent absorptive character. The light absorption range of Co-L/C_3_N_4_ reached 650 nm, while the band energy reduced to 2.30 eV, which caused a significant enhancement in photocatalytic activity. The conversion of substituted mandelic acids was more than 90% due to the photoactivity of Co-L/C_3_N_4_. Time-resolved PL spectra indicated the remarkable separation of the photogenerated electron–hole pairs in Co-L/C_3_N_4_. Furthermore, the UV-vis and *in situ* FTIR spectra indicated the formation of aldehyde groups in the selective oxidation process, which provided support for the plausible catalytic mechanism.

## Abstract 2

According to the requirements for sustainable development, reclaiming fine chemicals from wastewater under mild conditions is an extremely significant line of research.

## Graphical abstract

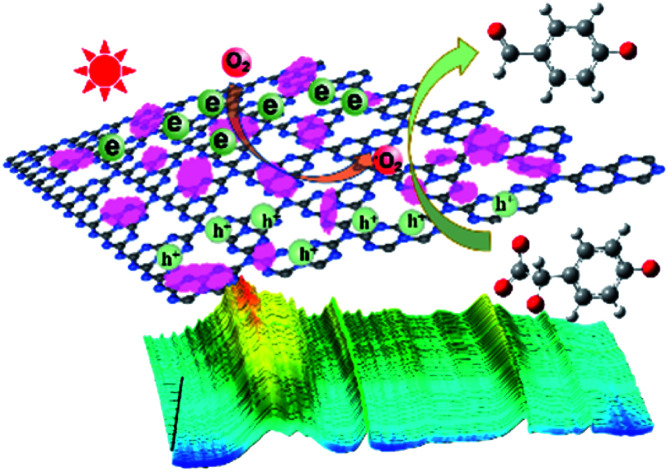

## 1 Introduction

In the last few decades, rapid industrial progress, especially in the fine chemicals industry, has dramatically increased the occurrence of chemical-induced water pollution. Mandelic acid and its derivatives (MADs) are such a series of chiral compounds that have attracted much attention due to their wide use in the synthesis of antibiotics, antiobesity and antitumor agents, biologically active compounds and enantiomer separation.^1,2^ Due to their superior water solubility, the concentration of MADs is substantial in relevant wastewater, causing serious environmental problems because of their strong negative environmental impact and high toxicity.^3^ Considering not only environmental pollution improvement but also resource re-utilization, converting MADs into useful chemicals, *i.e.* aromatic aldehydes, is a much more potent alternative to direct mineralization. Aromatic aldehydes, such as *p*-chlorobenzaldehyde, *p*-hydroxybenzaldehyde, 4-hydroxy-3-methoxybenzaldehyde, 4-hydroxy-3-ethoxybenzaldehyde, and 4-hydroxy-3-methoxy-5-methyl-benzaldehyde, have been used as pivotal intermediates in the pharmaceutical, printing and dyeing, pesticide, cosmetic, flame retardant and spice industries.^4–7^ Typically, *p*-hydroxybenzaldehyde is mainly used to synthesize the cardio-cerebrovascular medicine esmolol, the oral antibiotic amoxicillin, the antibacterial sulfa synergist trimethoxy benzylamine, the anti-hepatic fluke nitroiodophenol nitrile, among others.^8–11^

However, due to the outstanding water solubility of MADs, their highly efficient conversion has always been the focus of attention, especially under low MAD concentration.^12^ The conversion rate and aromatic aldehyde selectivity of MADs can reach 85% and 70–80%, respectively, when various nanoparticles, such as Bi(0), Co(ii), and Cu(ii), are employed to catalytically convert MADs in DMSO.^13–17^ During this process, the introduction of organic solvents causes potential environmental problems. Silica-encapsulated Cu–Al hydrotalcite (SECuAlHT) was developed as an efficient catalyst to catalytically oxidize 4-hydroxy-3-methoxymandelic acid in a.q. solution under mild conditions, which gave a 72% yield of 4-hydroxy-3-methoxybenzaldehyde.^18^ Without a doubt, the chemical oxidation efficiency towards the substrate was limited under low substrate concentration.

**Scheme 1:**
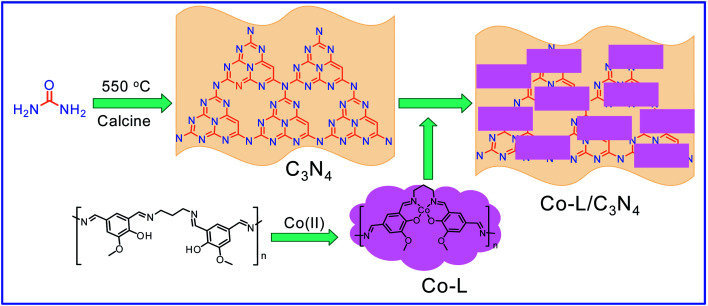
The synthetic process of the Co-L/C_3_N_4_ composite catalyst.

Considering that environmental pollution and the sustainable supply of green energy are the two main global challenges being faced in the current era, photocatalytic selective oxidation technology has become a focus of international attention as it has potential for development.^19–22^ Photocatalysis has been widely used in water splitting, the degradation of organic pollutants, CO_2_ conversion, selective organic synthesis, and so on.^23–26^ In order to reclaim useful products from wastewater with low energy consumption and high efficiency, photocatalytic technology has attracted extensive attention due to its advantages of clean and environmentally friendly character derived from the use of solar energy.^27^ Thanks to the advantageous non-polluting nature and low-energy consumption, heterogeneous photocatalysis has been widely used for the generation of clean energy and catalytic oxidation. Several aromatic aldehydes have been obtained from the corresponding alcohols by employing photocatalytic technology in the water medium at room temperature and atmospheric pressure.^28^ It has been widely used for the detoxification of water and the air and has become a very promising fine chemical synthesis method in recent years.^29^ Without a doubt, noble metal catalysts, such as Au, Ru, and Pd, can mediate efficient oxidation reactions due to the low-temperature or preferential oxidation of CO, alcohol oxidation and soot oxidation.^30^ However, high cost is the dominating disadvantage of noble metal-based catalysts, limiting their large-scale use. Thus, low-cost catalyst construction has immense potential. In recent decades, metal-free semiconductor polymer carbon nitride (C_3_N_4_) has attracted attention due to its unique and interesting physical and chemical properties, such as high working efficiency under visible and ultraviolet light, strong stability, non-toxicity, non-polluting nature.^31–34^ For further improvement of the photocatalytic activity of C_3_N_4_, many strategies have been adopted, including doping with different metals, such as palladium, silver, and cobalt.^35–38^ Based on the principles of green sustainable chemistry and engineering, considerable efforts have been made to adjust the catalytic activity of C_3_N_4_ toward achieving excellent conversion and selectivity in aqueous solutions.^39^ Simultaneously, metal–organic complexes have attracted the attention of scientists due to their excellent catalytic activity and tunable structure. Considering that C_3_N_4_ exhibits excellent electron-transition ability due to the π-conjugated system, supporting π-conjugated compounds on C_3_N_4_ has been used to develop hybrid photocatalysts for selective photocatalytic oxidation in the fields of water splitting and organic synthesis.^40,41^ Especially, the usage of industrial solid waste for organic ligand synthesis can further realize the resource utilization of industrial by-products. For example, 5-aldehyde vanillin (5-AV) is the typical by-product formed during the production of vanillin. The oxhydryl and aldehyde groups make it an abundantly tuneable structure (Scheme 1).

Herein, a polydentate chelate and polymeric metal–organic complex based on 5-AV, a by-product of vanillin, was constructed and doped on the surface of C_3_N_4_ (Co-L/C_3_N_4_) to achieve the selective conversion of a variety of substituted mandelic acids to aromatic aldehydes efficiently under mild photocatalytic conditions. The conversion of the substrate reached 96.3% with an 84% selectivity. More importantly, the plausible mechanism for aromatic aldehyde production was investigated by employing *in situ* IR and UV-vis spectroscopy.

## 2. Experimental details

### 2.1 Reagents and instruments

All materials were of AR grade. Melamine, CoCl_2_·6H_2_O, 1,3-diaminopropane, mandelic acid, 4-chloromandelic acid, 4-hydroxy-3-methoxymandelic acid, 4-hydroxy-3-ethoxymandelic acid, 4-hydroxy-3-methoxy-5-methylmandelic acid and ethanol were purchased from Sinopharm Chemical Reagent Co., Ltd., China. All the mentioned chemicals were directly used without additional purification. 5-Aldehyde vanillin was purchased from Jiaxing Zhonghua Chemical Co., Ltd.

### 2.2 Experimental method

#### 2.2.1 Preparation of Co-L/C_3_N_4_

The synthesis process of C_3_N_4_ was according to a previously reported method: melamine (0.1 mol, 12.6 g) was added to a ceramic crucible and heated to 550 °C at a ramping rate of 3 °C min^−1^ and held at 550 °C for 4 h. After cooling it to room temperature, 220 mg C_3_N_4_ was collected as a light-yellow solid.

Then, 5-aldehyde vanillin (5.5 mmol, 1.0 g) and 1,3-diaminopropane (5.5 mmol, 0.41 g) were completely dissolved in ethanol (30 mL) under vigorous stirring at 75 °C and refluxed for 4 h. After it cooled down to room temperature, the solvent was evaporated to give ligand (L) as a white solid. The ligand (219 mg, 1 mmol) and CoCl_2_·6H_2_O (238 mg, 1 mmol) were added to EtOH (10 mL) and heated to 80 °C for 3 hours. After they cooled down to room temperature, the solvent was removed in a vacuum to get the Co(ii) complex (Co-L). A certain amount of Co-L was mixed with C_3_N_4_ uniformly and calcined at 500 °C for 2 hours to obtain the Co/C_3_N_4_ catalyst (0.05 g Co-L mixed with 1 g C_3_N_4_ is denoted as Co-L/C_3_N_4_-5).

#### 2.2.2 Photocatalytic conversion performance of by Co-L/C_3_N_4_

The typical procedure for testing the photocatalytic conversion performance was as follows: the freshly prepared catalyst (Co/C_3_N_4_ 0.1 g) was added to an aqueous solution of mandelic acid (100 mL, 1.0%). After the pH value was adjusted to 11 using aqueous NaOH (30%), the mixture was bubbled with O_2_ under constant stirring at room temperature. Simultaneously, the mixture was irradiated with a 500 W Xe lamp for 5 hours, and the residual material was determined by HPLC. The pH value of the mixture was adjusted to 3.0–3.5 by using 6 M HCl. The mixture was washed with EtOAc (50 mL × 3). The combined organic layers were washed with brine, dried over MgSO_4_, filtered and concentrated to give the crude product. The residue was purified by silica gel chromatography (petroleum ether/EtOAc = 10 : 1 to 1 : 1) to give the product as a white solid (0.563 g, yield 77.6%). ^1^H-NMR (DMSO-d_6_, 600 MHz) *δ*: 9.39 (s, 1H), 7.19 (d, *J* = 12.00 Hz, 2H), 6.72 (d, *J* = 12.00 Hz, 2H), 5.62 (s, 1H), 4.90 (s, 1H). The structure and ^1^H NMR spectra of the products are shown in Fig. S1.†

#### 2.2.3 Characterization of the C_3_N_4_ and Co-L/C_3_N_4_

X-ray data from suitable single crystals were collected at 293 K on a Focus D8 (Bruker, Germany) with Cu Kα radiation (*λ* = 0.1542 nm). Fourier transform infrared spectroscopy (FT-IR) was performed by using a Thermo Nicolet IS5. The X-ray photoelectron spectroscopy (XPS) experiments were conducted on a Thermo Escalab 250 Xi system using Al-Kα radiation (*hν* = 1486.6 eV). The Brunauer–Emmett–Teller (BET) specific surface areas of the typical products were obtained at 77 K on a Micromeritics ASAP 2020 system. The UV-vis spectra were recorded on a UV-3900 spectrophotometer. A high-pressure mercury lamp (PLS-LAM500) and high-performance liquid chromatography (HPLC-Ulti Mate 3000, Thermo Fisher Scientific) were employed to detect material conversion. *In situ* ATR-FTIR spectroscopy was carried out on a Mettler Toledo Reactir-15.

## 3 Results and discussion

### 3.1 Excellent catalytic performance of the Co-L/C_3_N_4_ combined catalyst

**Fig. 1:**
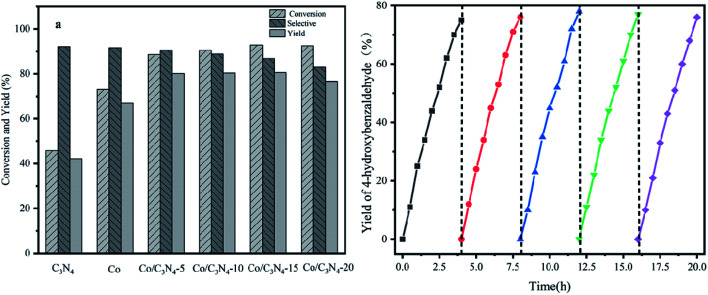
(a) The catalytic activity of 4-hydroxybenzaldehyde conversion with different Co-L/C_3_N_4_ ratios. (b) The yields of 4-hydroxybenzaldehyde under the catalytic action of Co-L/C_3_N_4_-10 over 5 continuous cycles.

As a representative, 4-hydroxyphenylglycolic acid (HPA) was employed as the model material to investigate the catalytic activity of the catalyst. In order to investigate the reaction clearly, the factors affecting the reactivity were considered one by one. Firstly, the catalytic activity caused by different catalyst loads was examined under typical conditions. As shown in Fig. 1a, in the presence of pure C_3_N_4_, the conversion ratio of HPA and the yield of 4-hydroxybenzaldehyde (HBD) were only 46.7% and 38.9%, respectively, indicating the mediocre catalytic activity of C_3_N_4_. When Co was loaded on pure C_3_N_4_ by employing an inorganic Co salt, such as CoCl_2_·6H_2_O, the conversion rate of HPA and the yield of HPB were 72.3% and 66.4%, respectively. This might be due to the agglomeration of the Co unit in the prepared catalyst. Co-L could be attached on the surface of C_3_N_4_ due to the π–π interactions, which avoided the agglomeration effectively. Furthermore, other types of Co sources were also introduced to be compared with Co-L-attached C_3_N_4_. As shown in Fig. S2,† the conversion of HPA ranged from 30% to 72% in the presence of other Co sources. With Co-L loaded on C_3_N_4_, the conversion of HPA and the yield of HBD increased rapidly, reflecting the excellent catalytic activity of Co-L/C_3_N_4_. The catalytic efficiency of Co-L/C_3_N_4_ was affected by the loading ratio of Co-L. As shown in Fig. 1a, HPA conversion increased from 87.8% to 94.0% when the Co-L loading amount increased from 5% to 20%. However, the highest yield of HBD reached was 77.6% in the presence of 10% Co-L/C_3_N_4_. This might be caused by the overoxidation of the obtained aldehyde in the presence of excess Co-L. Furthermore, the HPA conversion, HBD yield and selectivity corresponding to the repeated use of Co-L/C_3_N_4_-10 for 5 cycles are summarized in Fig. 1b. The photocatalytic activity of Co-L/C_3_N_4_-10 toward HBD formation hardly reduced after recycling over five runs, and the yield rate was maintained at 75%. Simultaneously, the catalyst was recovered after the reaction and characterized. The XRD and FT-IR, spectra, as well as TEM and SEM images, suggested that the catalyst underwent no obvious change compared with the freshly prepared catalyst, which indicated the superior stability and good photocatalytic performance of Co-L/C_3_N_4_-10 during the degradation process, as shown in Fig. S3† (Table 1)

**Table 1:** Comparison of the conversion of different substrates to corresponding aromatic aldehydes by Co-L/C_3_N_4_-10

Entry	Substrate	Product	Conv. (%)	Sel. (%)	Yield (%)
1	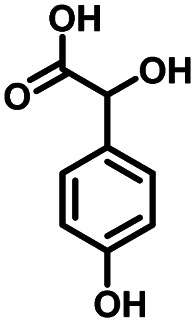	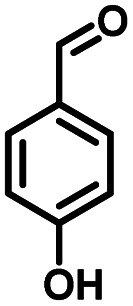	93.1	83.4	77.6
2	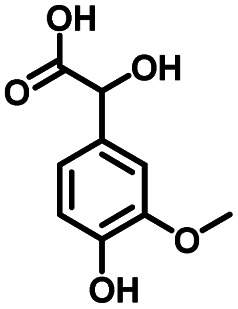	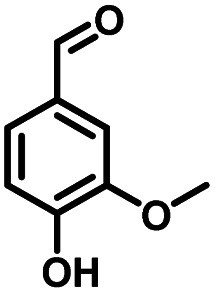	92.2	84.6	78
3	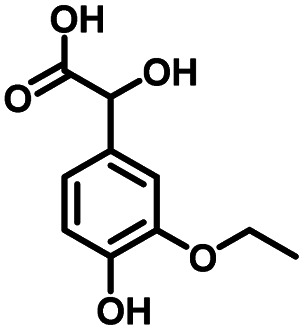	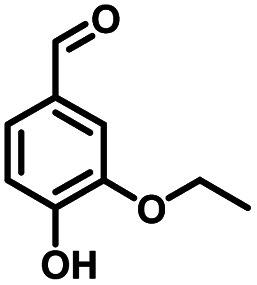	90.7	84.4	76.6
4	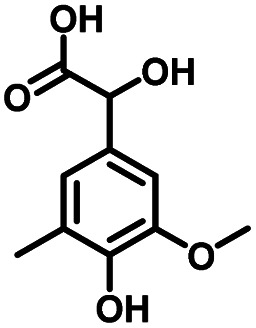	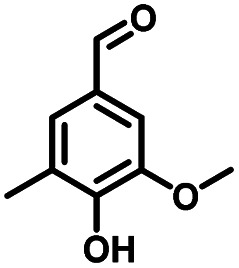	85.4	84.8	72.4
5	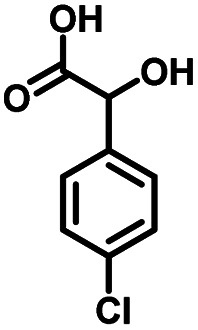	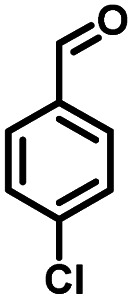	68.5	62.6	42.9

### 3.2 Characterization of the prepared Co-L/C_3_N_4_ composite catalyst

SEM and TEM were employed to investigate the morphology of the C_3_N_4_ catalyst. The obtained C_3_N_4_ presented a thin layered structure with folds that looked like a layer of tulle, confirming that it had a porous structure. Concurrently, the numerous mesopores on the surface of the Co-L/C_3_N_4_ composite catalyst displayed a structure with in-plane pores (as shown in Fig. S4†). As shown in Fig. S3c,† the chiffon-like structures with wrinkles and rippled edges were indexed to the C_3_N_4_ phase, and some large aggregates of irregular shape were evidently found in the field of vision, which suggested the possible coexistence of both phases and the coverage of C_3_N_4_ by the Co-L phase.

**Fig. 2:**
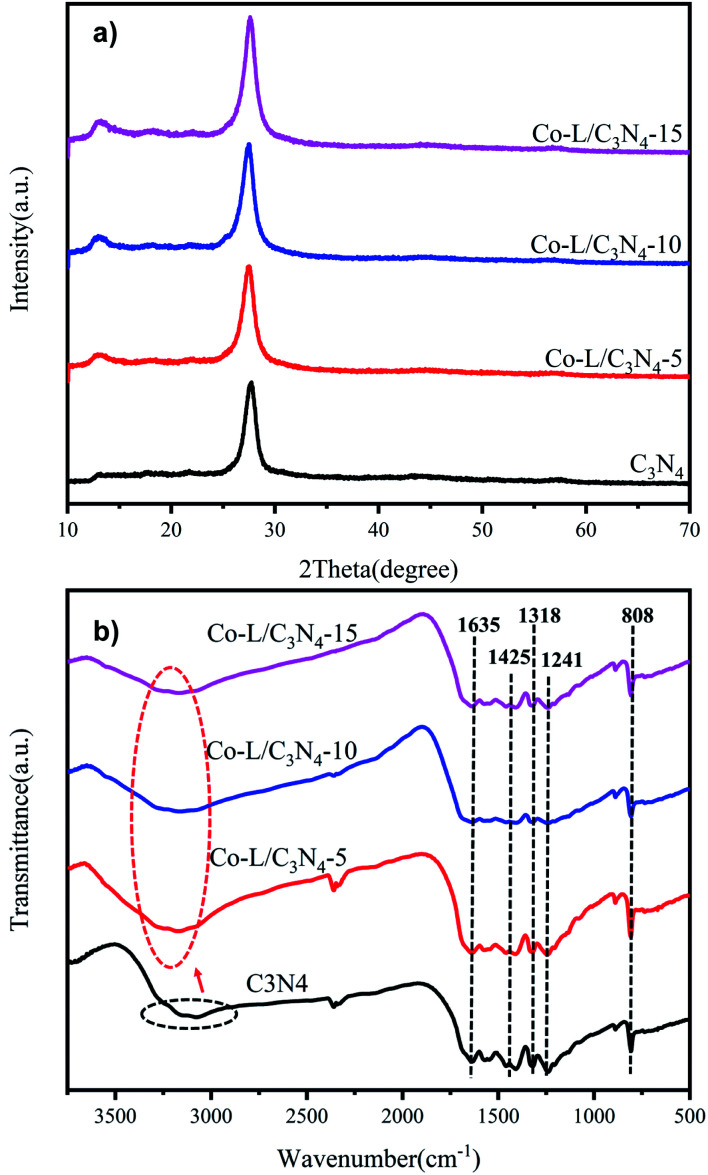
(a) X-ray diffraction (XRD) patterns of C_3_N_4_ and Co/C_3_N_4_; (b) FT-IR spectra of C_3_N_4_ and Co/C_3_N_4_.

The crystalline structures of C_3_N_4_ and Co-L/C_3_N_4_ were examined by X-ray diffraction (XRD). As shown in Fig. 2a, the peaks at 13.5° and 27.2° were ascribed to the reflections of the (100) and (002) planes of C_3_N_4_, respectively.^42^ Interestingly, the peaks at 13.5° and 27.2° barely appeared in Co-L/C_3_N_4_, and these peaks became sharper and more obvious with the gradual increase in the Co-L ligand content, reflecting the enhanced crystallinity of the Co-L/C_3_N_4_ structure.^36,43^ The XRD results further confirmed the successful preparation and high stability of the Co-L/C_3_N_4_ heterostructure.^44^ In addition, the FT-IR spectra of the C_3_N_4_ and Co-L/C_3_N_4_ samples (Fig. 2b) exhibited some strong absorption peaks at 808 cm^−1^, which was the characteristic vibration of the out-of-plane bending of the triazine rings. The peaks at 1200–1650 cm^−1^ were attributed to the C–N and C=N heterocycles, and those between 3000–3500 cm^−1^ could be assigned to the N–H/O–H groups. Interestingly, Co-L/C_3_N_4_ had a characteristic band similar to C_3_N_4_, indicating that the typical graphite structure of the carbonitride was not destroyed after Co-L doping. However, the peak at around 3100–3400 cm^−1^ was much narrower than that in the C_3_N_4_ spectrum, while the intensity was much greater, which is completely consistent with the description in the literature.^45–47^

**Fig. 3:**
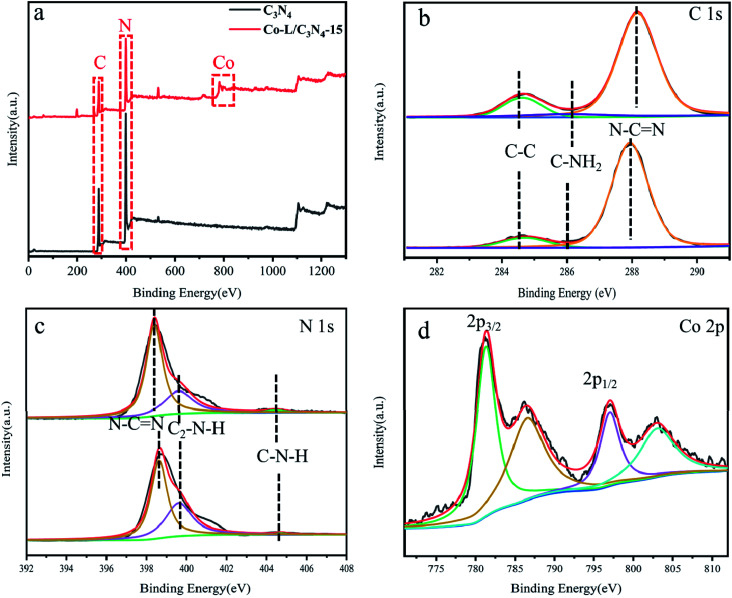
(a) X-ray photoelectron spectroscopy (XPS) of C_3_N_4_ and Co-L/C_3_N_4_-15; (b–d) high-resolution XPS spectra of C 1s, N 1s and Co 2p.

To further investigate the surface microstructure of Co-L on the C_3_N_4_ nanoparticles, XPS (X-ray photoelectron spectroscopy) characterization was employed to investigate the constitution of the C_3_N_4_ and Co-L/C_3_N_4_-10 photocatalysts. As shown in Fig. 3a, both the C_3_N_4_ and Co-L/C_3_N_4_ samples consisted of C and N elements. Furthermore, a peak for Co appeared in the Co-L/C_3_N_4_ photocatalyst. In the high-resolution XPS analysis, the C 1s (Fig. 3b) deconvoluted peaks were centred at 284.5, 286.2 and 288.1 eV.^48,49^ The peak at 284.5 eV was ascribed to the physically adsorbed carbon species or the sp^2^ C–C bonds, and the peak at 286.2 eV was assigned to the C–NH_2_ species, while the strong peak at 288.1 eV could be attributed to N–C=N. The three peaks at binding energies 398.6, 400.4 and 404.5 eV could be attributed to the triazine rings N–C=N, C_2_–N–H and C–N–H, respectively (as shown in Fig. 3c), which belong to the typical N 1s peaks in C_3_N_4_.^50–53^ In the high-resolution XPS analysis, the Co 2p peak at (Fig. 3d) the binding energy of 781.2 and 796.2 eV could be attributed to the Co(ii) state in the form of Co–N. This indicated that the valence state of the Co ion in Co-L/C_3_N_4_ was Co(ii) rather than Co(iii). As shown in Fig. S5,† the Co 2p spectrum could be divided into Co^2+^ and Co^3+^ fitting curves, and four shakeup satellites. The fitting peaks at 782.5 and 797.4 eV could be grouped to Co^3+^, whereas the peaks at 779.5 and 794.5 eV were assigned to Co^2+^.^38,54–56^

**Fig. 4:**
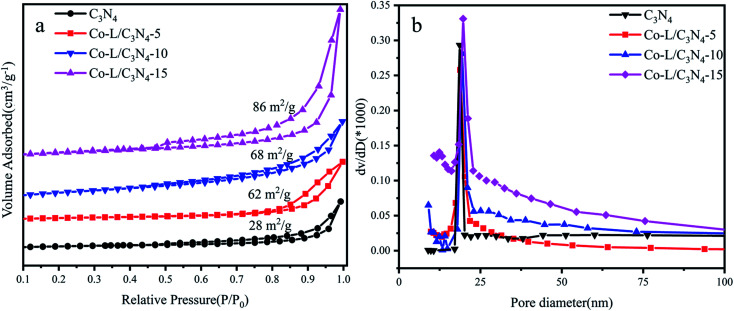
(a) N_2_ adsorption–desorption isotherms of C_3_N_4_ and Co-L/C_3_N_4_; (b) the corresponding pore size distribution curves of C_3_N_4_ and Co-L/C_3_N_4_.

The N_2_ adsorption–desorption isotherms and pore size distribution curves of C_3_N_4_ and Co-L/C_3_N_4_-10 were recorded by the BET method, as shown in Fig. 4. All the prepared samples exhibited typical type IV isotherms and H3 hysteresis loops (Fig. 4a), implying the remarkable mesoporous properties of the materials.^57^ As a typical porous material, the specific surface area of C_3_N_4_ was 28 m^2^ g^−1^. As expected, the specific surface area of all the catalysts increased from 28 m^2^ g^−1^ to 86 m^2^ g^−1^ following the addition of 15% Co-L. The increase in specific surface area after Co doping was mainly due to the rich pore structure. As shown in Fig. 4b, the pore size and pore volume of the Co-L/C_3_N_4_ catalyst were larger than those of C_3_N_4_.^58^

**Fig. 5:**
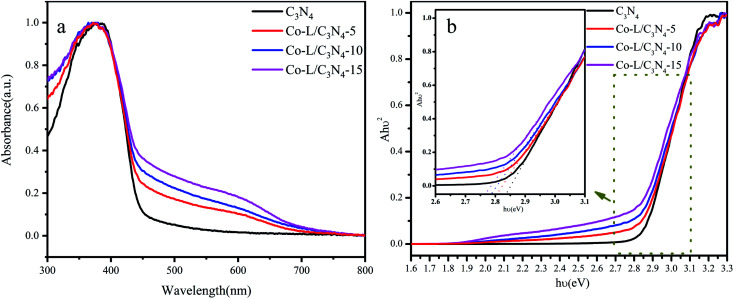
(a) The UV-visible diffuse reflectance spectra of C_3_N_4_ and Co-L/C_3_N_4_; (b) the bandgap energy of C_3_N_4_ and Co-L/C_3_N_4_.

The optical properties of the as-prepared photocatalysts were characterized by UV-vis diffuse reflectance spectroscopy (DRS). The DRS curves of the as-prepared C_3_N_4_ and Co-L/C_3_N_4_ samples are shown in Fig. 5a; C_3_N_4_ showed an absorption edge at ∼450 nm, suggesting its limited photo-response to visible light. More importantly, the absorption edge of Co-L/C_3_N_4_ was obviously red-shifted in comparison with C_3_N_4_.^59^ The optical absorption intensity of Co-L/C_3_N_4_ was significantly enhanced in the range of 550–650 nm. These results describe that Co doping facilitates secondary absorption in the visible light region, which would promote absorption in the wider range of visible light and the absorption efficiency of the system, resulting in higher photocatalytic activity.^60,61^ To further investigate the effect of Co doping on the optical bandgap energy of Co-L/C_3_N_4_, the Kubelka–Munk method was used to calculate the bandgap of the prepared samples.^62^ As shown in Fig. 5b, the presence of the Co-L ligand slightly reduced the energy band of C_3_N_4_. The results show that the Co-L ligand presented a good optical response in the whole visible wavelength range (*E*_g_ of Co-L was about 1.85 eV, as shown in Fig. S6†). At the same time, the optical response intensity of the Co-L/C_3_N_4_ hybrid material was significantly stronger than that of ordinary C_3_N_4_. With an increase in Co-L ligand content, the fluorescence intensity increased gradually, especially in the range of 550–650 nm. This phenomenon showed that the introduction of Co-L effectively improved the light absorption ability of the C_3_N_4_ material.^63^

**Fig. 6:**
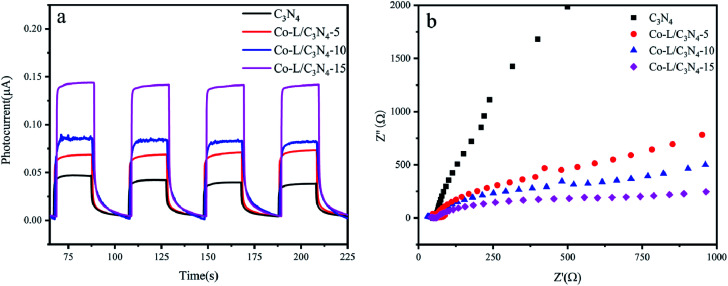
(a) Transient photocurrent response of C_3_N_4_ and Co-L/C_3_N_4_; (b) electrochemical impedance spectra of C_3_N_4_ and Co-L/C_3_N_4_.

As shown in Fig. S7,† the Mott–Schottky plot was obtained in aqueous solutions containing 0.1 M KCl at pH = 7.0 to estimate the EFB of Co-L and C_3_N_4_, respectively. The values of EFB were estimated to be −0.86 V and −0.97 (*vs.* NHE) for Co-L and C_3_N_4_, respectively. Compared with C_3_N_4_, a positive shift in the EFB of Co-L demonstrated a decrease in the bending of the band edge, thus facilitating electron transfer.^64^ The reason for the improvement in the photocatalytic activity of the Co-L/C_3_N_4_ nanocomposites was investigated by photochemical experiments. As shown in Fig. 6a, the photocurrent responses of C_3_N_4_ were much lower than that of Co-L combined C_3_N_4_ due to the rapid recombination of the photoexcited e^−^ and h^+^. However, the photocurrent intensity increased dramatically along with the introduction of Co-L, and the Co-L/C_3_N_4_-15 catalyst exhibited the highest photocurrent density, which is consistent with the steady-state PL results. The obvious changes in the current response of the Co-L/C_3_N_4_ samples under irradiation indicated the good photo-harvesting capability of the prepared samples. Moreover, after several switching cycles, the photo-response was basically stable, further indicating the strong light corrosion resistance of the nanocomposites.^65,66^ At the same time, it was observed that the transient current between the lamps decreased, which may be caused by the accumulation of charges before the lamp is turned on. The photoelectric response was closely related to the content of Co ions. The photocurrent of Co-L/C_3_N_4_-15 was 3 times that of C_3_N_4_, which indicated the excellent photo-response of the composite nanomaterial. For nano-photocatalysts, reducing the interfacial resistance can promote charge transfer and thus improve photocatalytic performance.^67^ The interfacial properties of semiconductors are usually studied by electrochemical impedance spectroscopy (EIS).^68^ The interfacial resistance of the sample is expressed by the radius of the semicircle in the EIS.^69^ As shown in Fig. 6b, the radius of the C_3_N_4_ curve is larger than those of the Co-L loaded catalysts, and the radius of the semicircle decreases obviously after the introduction of Co ions, indicating that the addition of Co-L clearly reduces the resistance at the C_3_N_4_ interface. The interface of the Co-L/C_3_N_4_-15 composite nanomaterials had minimum resistance, which is consistent with the results of the photocurrent.

**Fig. 7:**
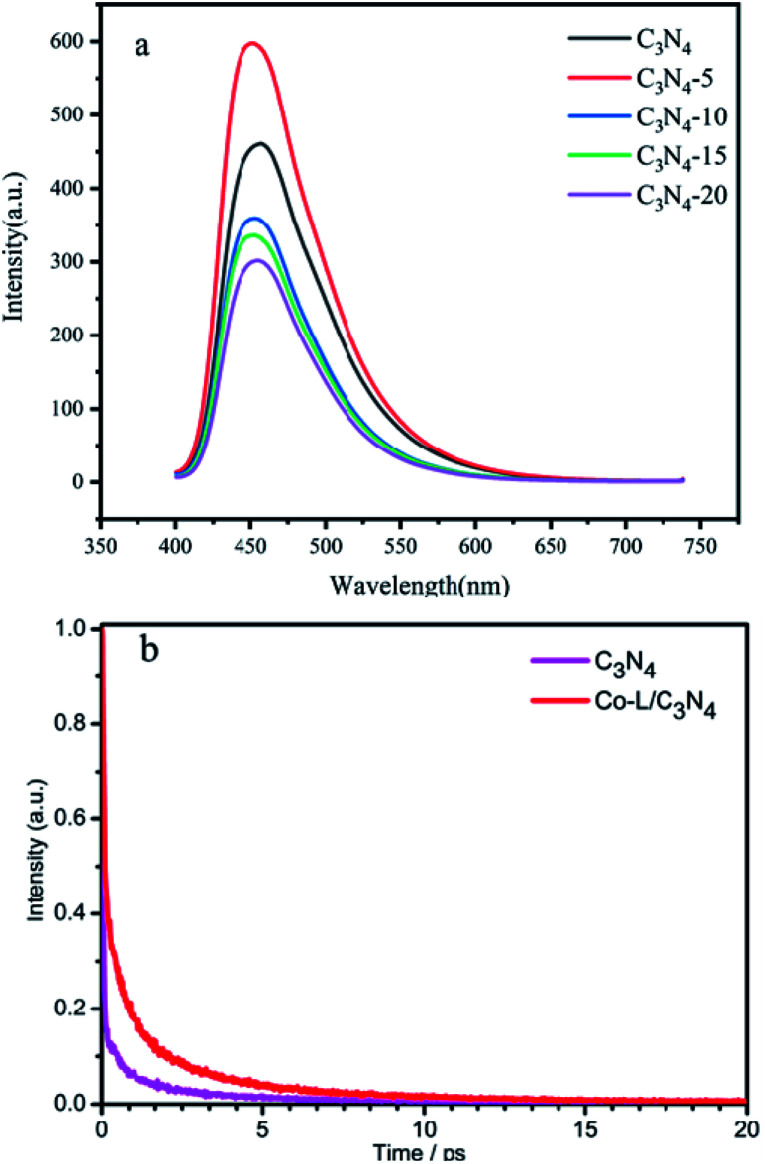
(a) The PL spectra of C_3_N_4_ and different ratios of Co-L/C_3_N_4_. (b) The transient fluorescence spectra of C_3_N_4_ and Co-L/C_3_N_4_.

Fluorescence spectroscopy is an effective method to investigate the efficiency of photogenerated electron–hole separation in semiconductors.^70^ The fluorescence spectra of C_3_N_4_ and Co-L/C_3_N_4_ were measured at the same excitation wavelength (*λ*_ex_ = 390 nm). As shown in Fig. 7a, C_3_N_4_ showed a strong fluorescence peak near 455 nm. After Co-L was loaded on C_3_N_4_, the fluorescence peak intensity of Co-L/C_3_N_4_ decreased significantly, indicating effectively suppressed recombination of the photogenerated charges. Furthermore, the photogenerated electron–hole recombination is also the critical factor to improving the photocatalytic oxidation efficiency. As shown in Fig. 7b, to further study the behaviour of the charge carriers in the samples, the time-resolved PL spectra were recorded, and the results are shown in Fig. 7b. C_3_N_4_ afforded a shorter average fluorescence lifetime (3.07 ps) compared with the Co-L/C_3_N_4_ (5.79 ps), which fully implied that the activated state in Co-L/C_3_N_4_ was long-lived than that in C_3_N_4_. The longer lifetime of electrons could be attributed to the remarkable separation of the photogenerated electron–hole pairs.^71–73^

### 3.3 Probable mechanism of aromatic aldehyde production by Co-L/C_3_N_4_

In order to investigate the mechanism of 4-hydroxyphenylglycolic acid oxidation, the probable reaction pathway was explored by employing *in situ* FTIR and UV absorption spectroscopy.

**Fig. 8:**
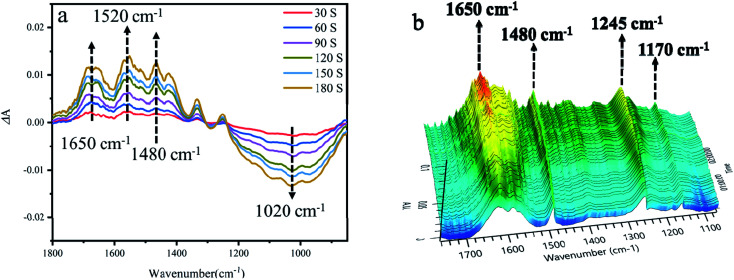
(a) The 2D *in situ* FTIR spectra of 4-hydroxyphenylglycolic acid oxidation under irradiation. (b) The 3D *in situ* FTIR spectra of 4-hydroxyphenylglycolic acid oxidation under irradiation.

The IR spectra of 4-hydroxybenzaldehyde and 4-hydroxyphenylglycolic acid in water were collected from a series of undersaturated solutions with known solute concentrations. To exhibit the reaction clearly, the differential absorption (Δ*A*) data was collected and processed, as shown in Fig. 8a. Peaks pointing down indicated bands disappearing, while those pointing up corresponded to new bands appearing due to the catalytic reaction. The increased intensity of the Δ*A* value at 1650 cm^−1^ was vested to the band of C=O stretching vibration, which represents the generation of 4-hydroxybenzaldehyde by oxidation of benzyl alcohol in 4-hydroxyphenylglycolic acid.^74^ At the same time, the increased Δ*A* peaks at 1245 and 1170 cm^−1^ (as shown in Fig. 8b) belonged to the C–C stretching vibration and the conjugation of aromatic ketones following the 4-hydroxybenzaldehyde ketonic acid generation.^75^ The peaks at 1520 cm^−1^ and 1480 cm^−1^ were attributed to the intermediates of the conversion of 4-hydroxyphenylglycolic acid to ketonic acid. The peak at 1020 cm^−1^ was attributed to the C–C stretching vibration. The decline in the peaks was attributed to the probable fracture of C–C caused by the decarboxylation of the intermediate ketonic acid.^76^ UV-visible spectroscopy was an important method to study the oxidation of 4-hydroxymandelic acid to 4-hydroxybenzaldehyde. As shown in Fig. S8,† the decrease in the strong absorption peak at 249 nm could be attributed to the π–π* transition of the benzene ring. The absorption peak at 332 nm was attributed to the n–π* transition of the aldehyde oxygen atom. The changes in the UV-visible spectrum indicated the transformation of 4-hydroxymandelic acid to 4-hydroxybenzaldehyde clearly under irradiation.

**Fig. 9:**
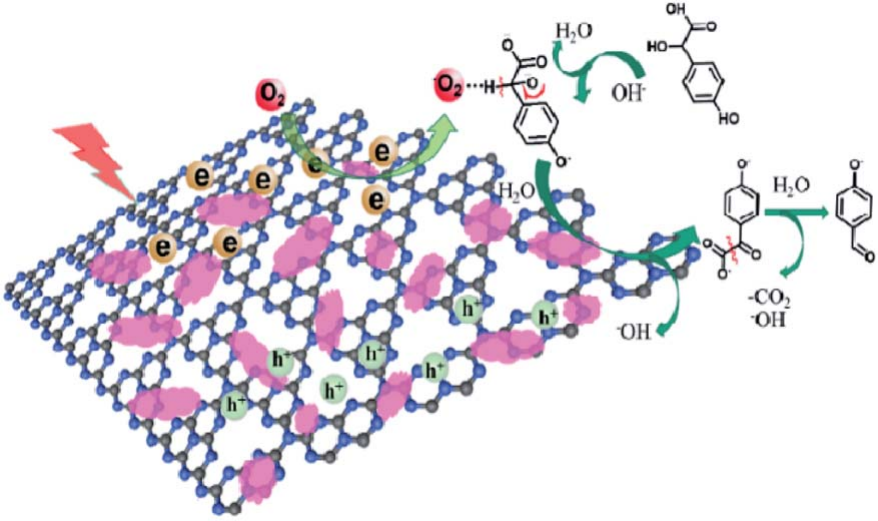
Proposed mechanism for *p*-hydroxymandelic acid photo-oxidation by Co-L/C_3_N_4_.

On the basis of the above results, a plausible mechanism for the catalytical process has been proposed, as shown in Fig. 9. The Co-L loaded to C_3_N_4_ significantly enhances the visible light absorption, which has been proven by the UV-Vis and PL valence band spectra analysis. Therefore, under visible light irradiation, electrons are excited to the conduction band from the valence band of C_3_N_4_, with the holes staying on the valence band simultaneously.^77–79^ The electrons in the conduction band of Co-L/C_3_N_4_ can be trapped by electrophilic O_2_ to produce superoxide radical anions (˙O_2_^−^).^80,81^ On the other hand, the transition of Co^2+^/Co^3+^ ensures the generation of ˙O_2_^−^, which causes the substrate oxidation reaction. The high concentration of ˙O_2_^−^ is captured by *p*-hydroxymandelic acid and further experiences the hydroxylation process to give OH^−^ finally. The deprotonated *p*-hydroxymandelic acid forms the pivotal intermediate (α-keto carboxylate radical) to accomplish the oxidation process. Simultaneously, *p*-hydroxybenzaldehyde is generated following the decarboxylation of the pivotal intermediate. The continuously generated ˙O_2_^−^ radicals greatly promote the conversion rate of *p*-hydroxymandelic acid in the photocatalytic process.

## 4 Conclusions

In conclusion, a Co-L/C_3_N_4_ composite photocatalyst was designed and prepared for high-efficiency aromatic aldehyde generation under normal temperature and pressure, with oxygen as the oxidant. The Co-L-loaded C_3_N_4_ could accelerate the separation of the photogenerated carriers and significantly improve photocatalytic activity. Under ultraviolet light irradiation, the conversion of *p*-hydroxybenzaldehyde was 93.1%, which is 2 times higher than that achieved with pure C_3_N_4_. The enhanced photocatalytic activity is attributed to the synergistic effect of Co-L and C_3_N_4_, which promote the remarkable separation of the photogenerated electron–hole pairs and charge transfer. This work provides a useful strategy for aromatic aldehyde production in wastewater containing substituted mandelic acid derivatives, thus promoting the recycling of waste resources.

## Conflicts of interest

There are no conflicts to declare.

## Supplementary Material

RA-012-D1RA08256F-s001
