# Supplementary material for: Solar-driven aromatic aldehydes: green production from mandelic acid derivatives by a Co(ii)/C3N4 combined catalyst in aqueous media
Source: RSC Adv. 2022 Feb 14;12(9):5245–54. doi: 10.1039/d1ra08256f (PMC8981277; doi:10.1039/d1ra08256f)
Supplement: RA-012-D1RA08256F-s001 [file RA-012-D1RA08256F-s001.pdf]

Electronic Supplementary Material (ESI) for RSC Advances.
This journal is © The Royal Society of Chemistry 2022

Supporting Information

Solar-driven Aromatic Aldehydes Green Production from Mandelic Acid Derivatives by Co(II)/C_3N_4 Combined Catalyst in Aqueous Media

Mi Wu, Hongzhao Wang[1], Haifang Mao[*1], Chaoyang Wang[1], Zhenbiao Dong[1], Ting Tang[*2], Wei Zheng[2], Lehong Jin[2], Jibo Liu[1*]

1. *School of Chemical and Environmental Engineering, Shanghai Institute of Technology, 100 Haiquan Road, 201418, Shanghai, China.*
2. *College of Medicine, Hangzhou Normal University, 2318 Yuhangtang Road, 311121, Hangzhou, Zhejiang, China.*

Table. S1 The Summering of the DFT Result

Compound	Charge for C_{Bn}	Energy (J/Mol)	HOMO	LUMO	HOMO	LUMO
1	0.132659	290924.7	8.642	11.506	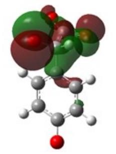	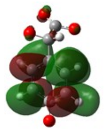
2	0.129555	373513.0	8.529	9.332	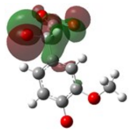	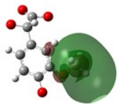
3	0.127283	445978.0	8.067	8.511	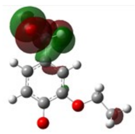	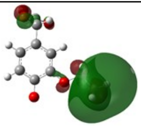
4	0.127803	443912.1	8.460	9.239	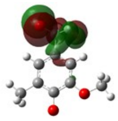	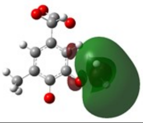
5	0.113843	286630.9	4.769	6.516	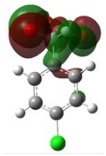	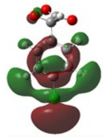

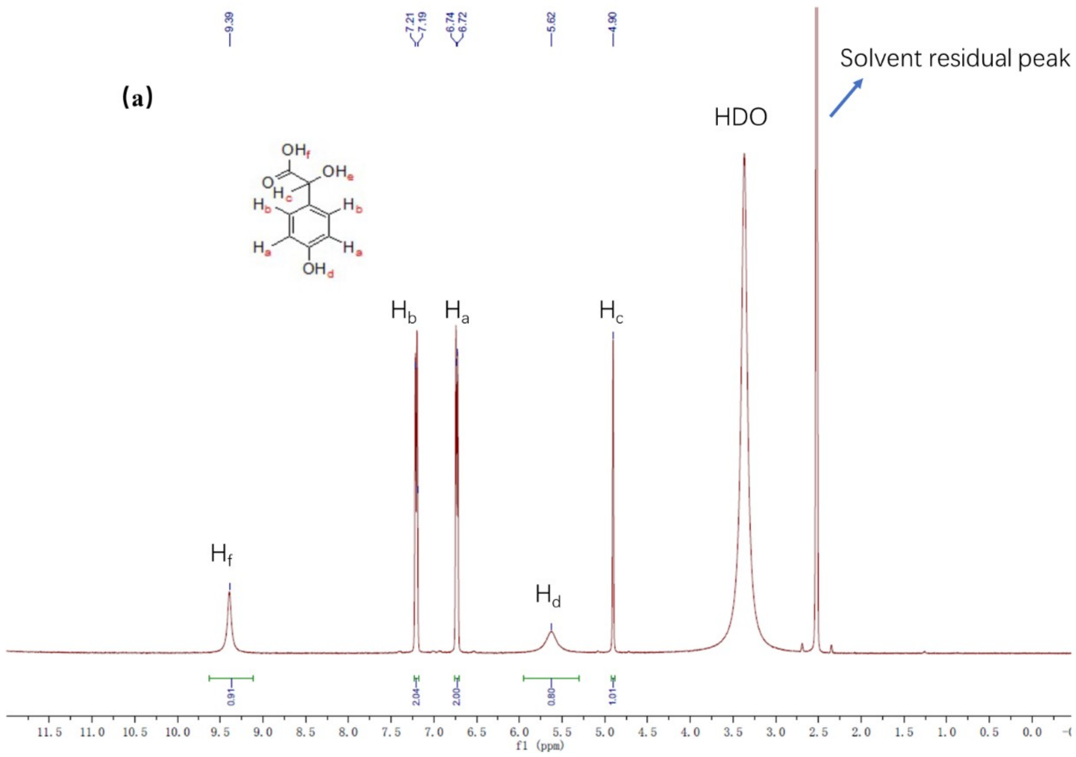

^{1}H-NMR (DMSO-*d₆*, TMS, 600MHz) δ: 9.39 (s, 1H), 7.19 (d, *J*=12.00 Hz, 2H), 6.72 (d, *J*=12.00 Hz, 2H), 5.62 (s, 1H), 4.90 (s, 1H).

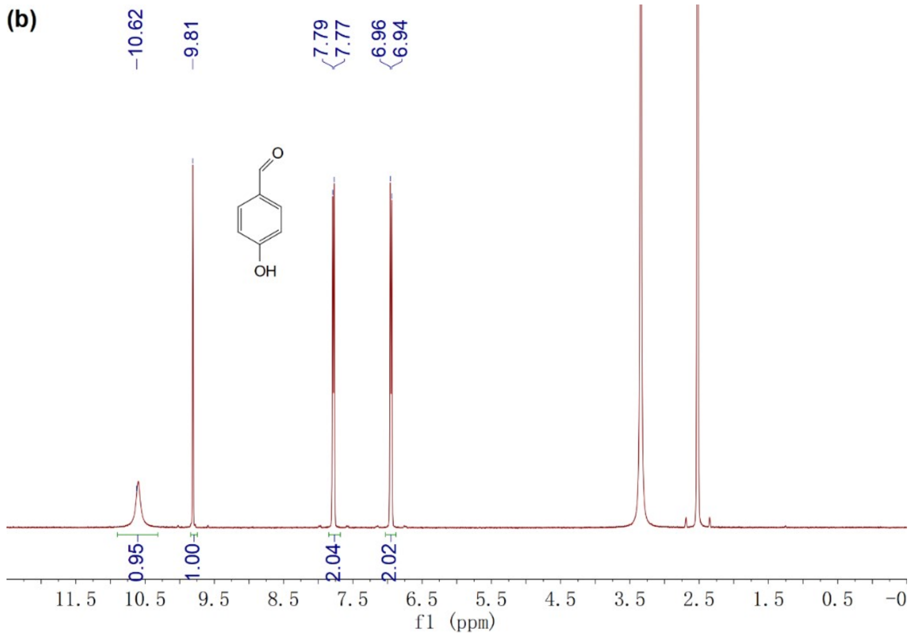

^{1}H-NMR (DMSO-*d6*, TMS, 600MHz) δ: 10.60 (s, 1H), 9.81 (s, 1H), 7.77 (d, *J*=12.00 Hz, 2H), 6.96 (d, *J*=12.00 Hz, 2H)

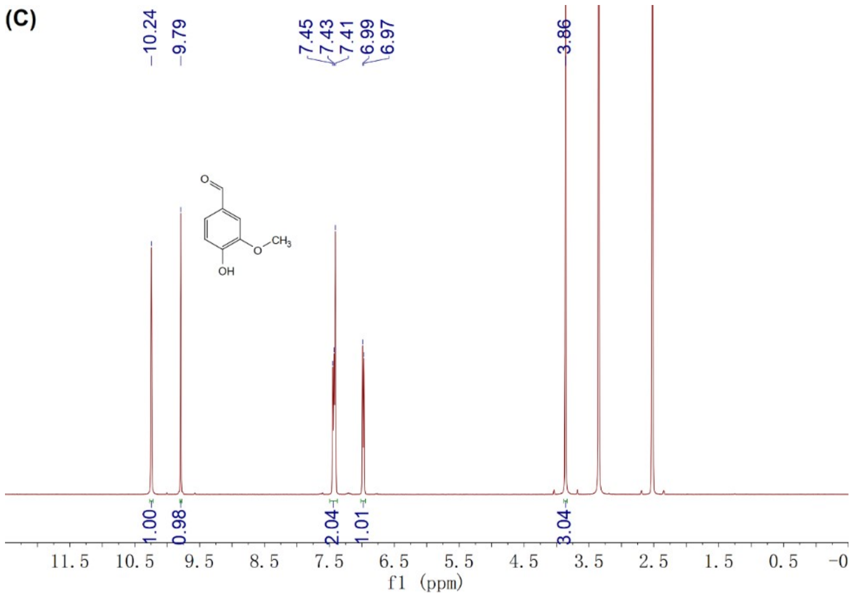

^{1}H-NMR (DMSO-*d6*, TMS, 600Hz) δ: 10.28 (s, 1H), 9.78 (s, 1H), 7.43 (t, J_1=12.00 Hz, J_2=12.00 Hz, 2H), 6.97 (d, 1H), 3.85 (s, 3H)

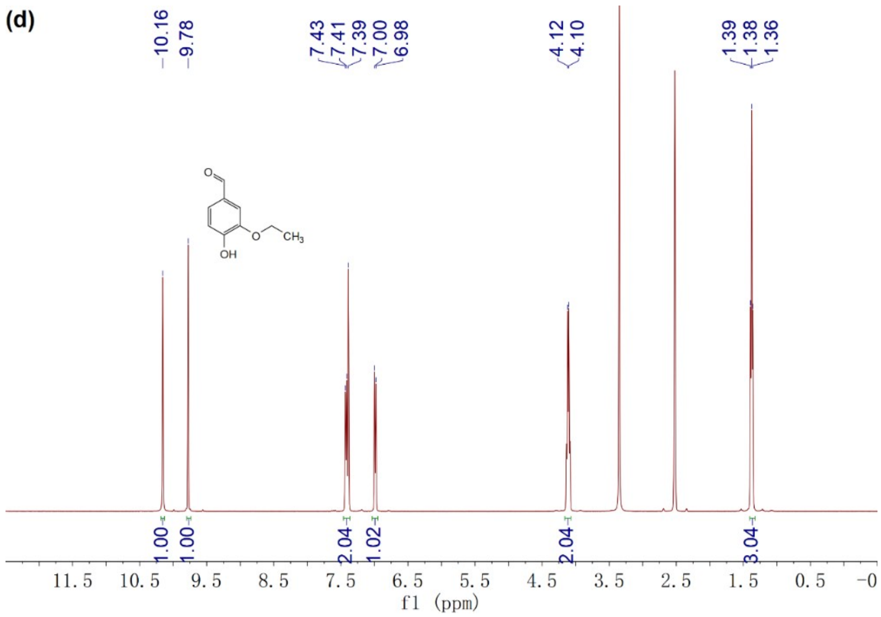

^{1}H-NMR (DMSO-*d6*, TMS, 600MHz) δ: 10.16 (s, 1H), 9.78 (s, 1H), 7.37 (t, *J* =12.00 Hz, 2H), 6.98 (d, *J* = 12.00 Hz, 1H), 4.07 (d, *J* = 12.00 Hz, 2H), 1.38 (t, J_1=12.00 Hz, 3H)

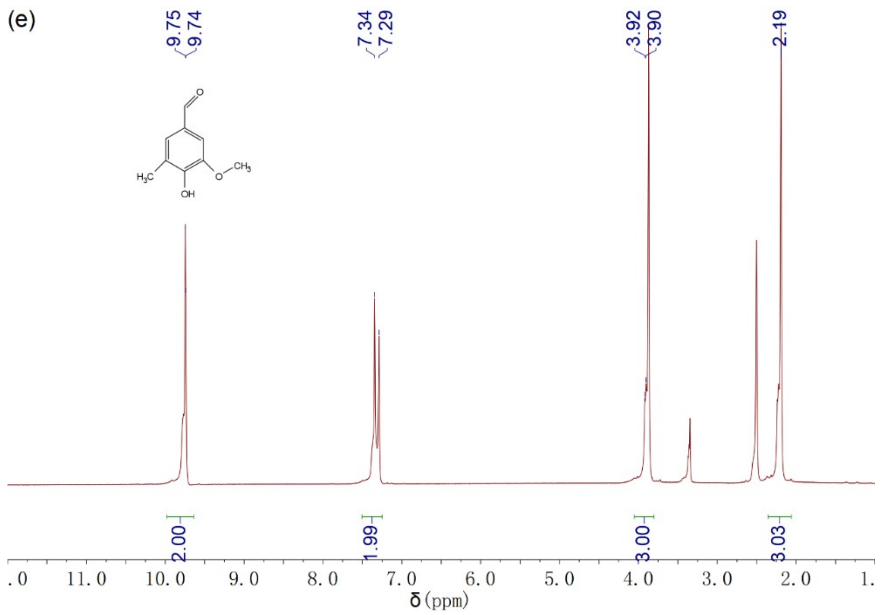

^{1}H-NMR (DMSO-*d*6, TMS, 600MHz) δ: 9.75 (s, 1H), 9.74 (s, 1H), 7.34 (s, 1H), 7.29 (s, 1H), 3.90 (s, 3H), 2.19 (s, 3H)

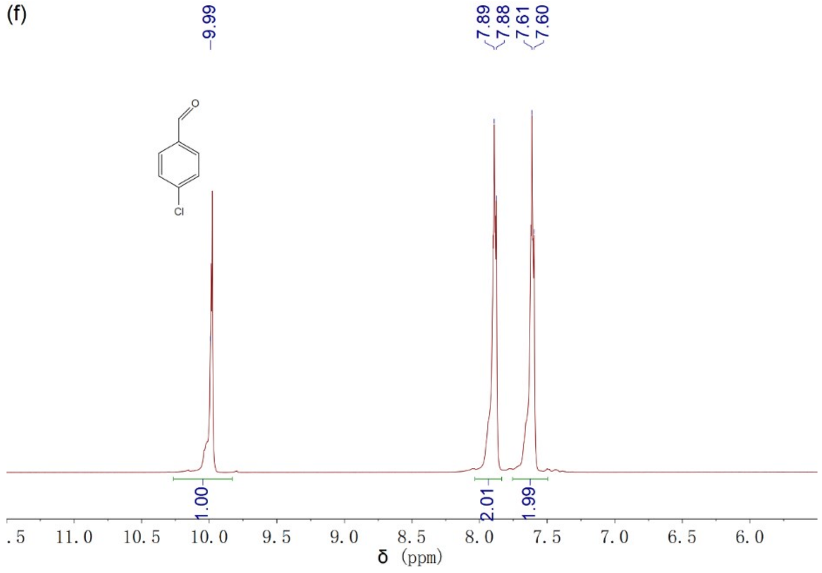

^{1}H-NMR (DMSO-*d*6, TMS, 600MHz) δ: 9.99 (s, 1H), 7.89 (d, *J*=6.00 Hz, 2H), 7.60 (d, *J*=6.00 Hz, 2H)

Fig. S1 *The ^{1}H-NMR spectra of 4-Hydroxyphenylglycolic acid (a), 4-hydroxybenzaldehyde (b), (c) 4-hydroxy-3-methoxymandehyde, (d) 4-hydroxy-3-ethoxymandehyde, (e) 4-hydroxy-3-methoxy-5-methylmandehyde and (f) 4-chlorosylmandehyde.*

Fig. S2 The catalytic efficiency of different Co source.

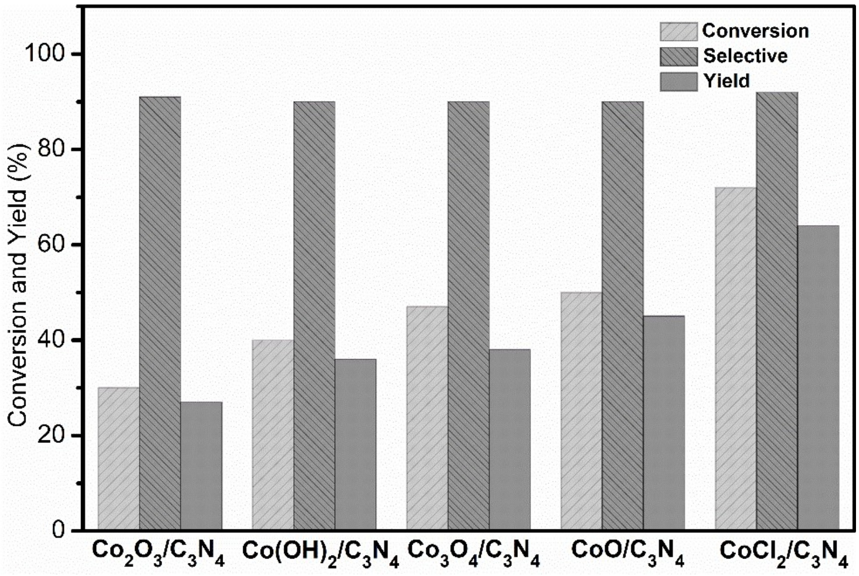

Fig. S3 (a) The field emission scanning electron microscopy (FESEM) image of freshly prepared Co-L/C_3N_4-10; (b) The field emission scanning electron microscopy (FESEM) image of Co-L/C_3N_4-10 after using; (c) The transmission electron microscopy (TEM) image of freshly prepared Co-L/C_3N_4-10; (d) The transmission electron microscopy (TEM) image of Co-L/C_3N_4-10 after using; (e) X-ray Diffraction of Co-L/C_3N_4-10 before after using; (f) The IR spectra of Co-L/C_3N_4-10 before after using.

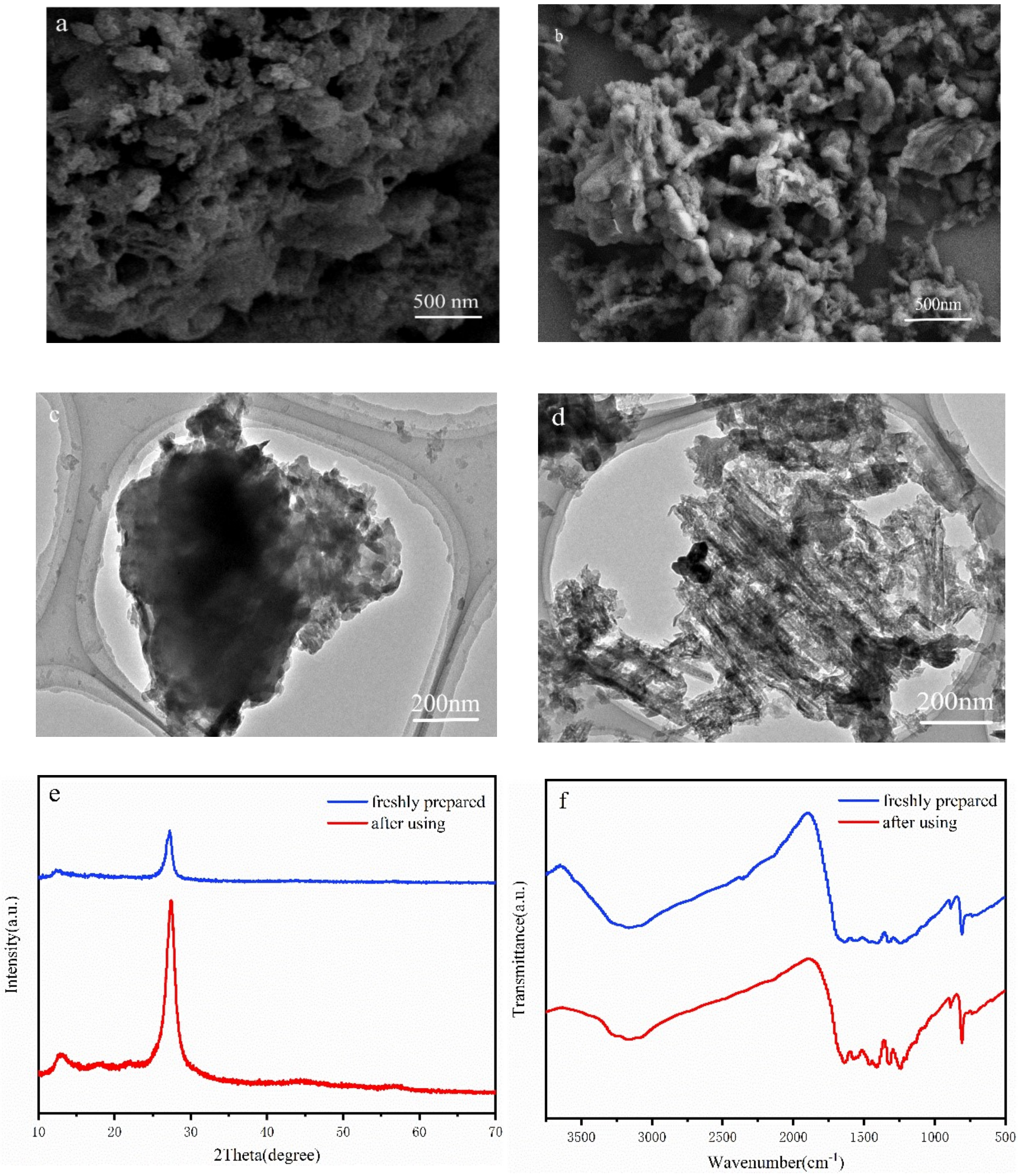

Fig. S4 (ab) The field emission scanning electron microscopy (FESEM) image of Co-L/C_3N_4; (cd) The field emission scanning electron microscopy (FESEM) image of C_3N_4.

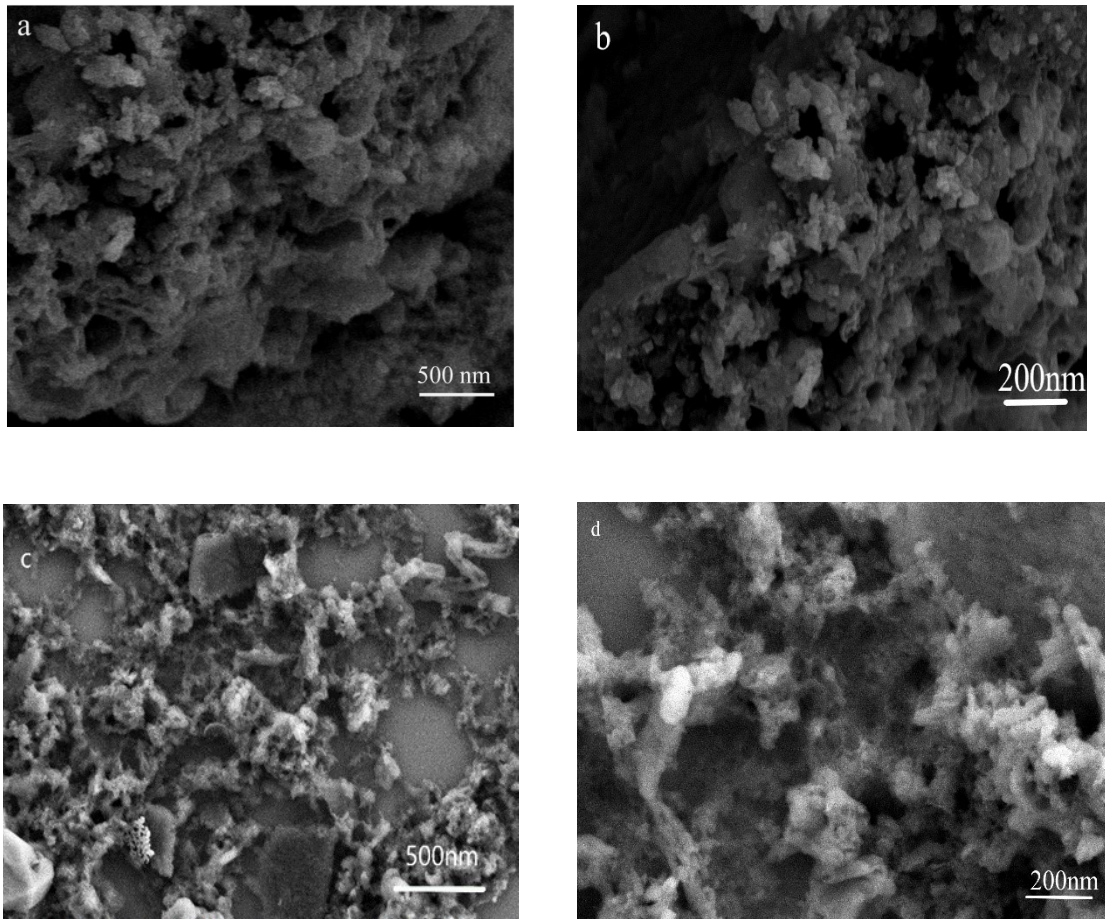

Fig. S5 X-ray photoelectron spectroscopy (XPS) of Co 2p in Co-L/C_3N_4-10 after 3 circles.

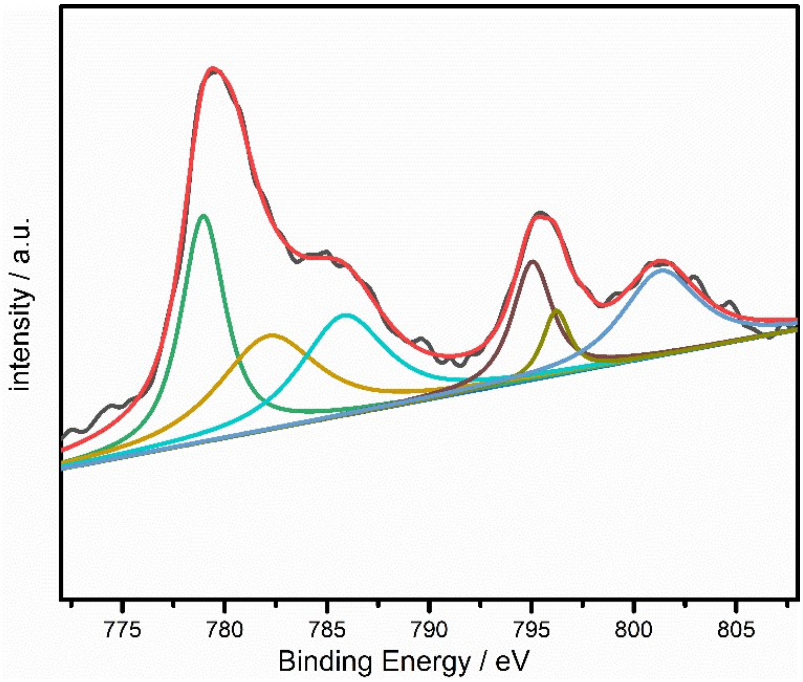

Fig. S6 (a)The UV-vis diffuse reflectance spectroscopy of Co-L. (b) The estimation of band gap energy of Co-L.

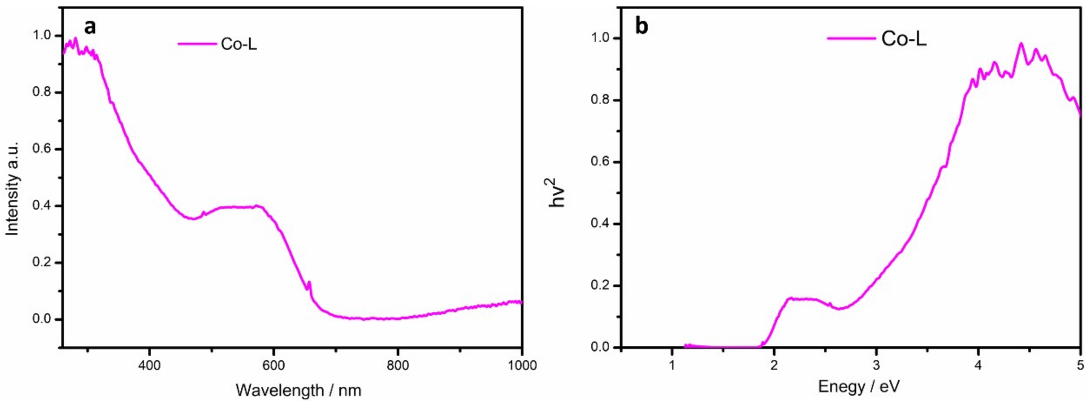

Fig. S7 The Mott-Schottky plot of C_3N_4 and Co-L at a frequency of 962 Hz in the dark, 0.1 M KCl aqueous solution as a supporting electrolyte, 50 mV s^{-1} scan rate, Co-L or C_3N_4 loaded FTO electrode as a working electrode (area: 0.25 cm^2), Ag/AgCl (filled with saturated KCl aqueous solution) as a reference electrode, Pt wire as a counter electrode.

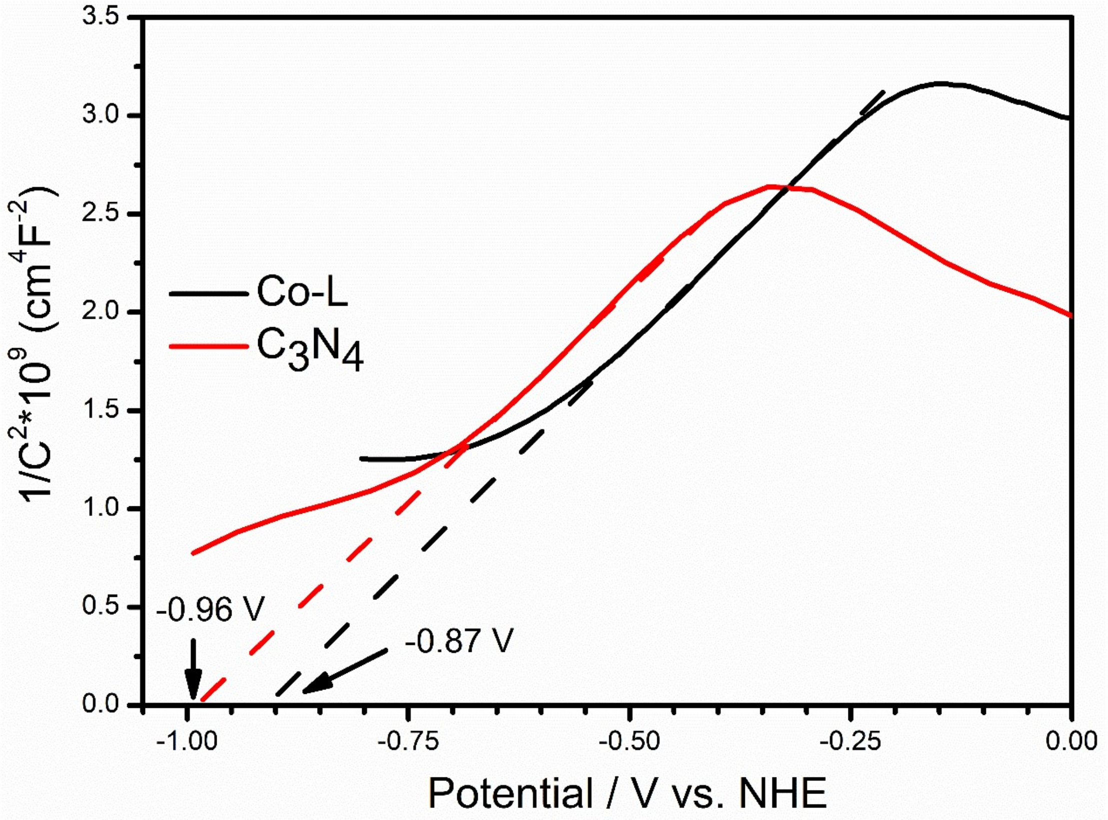

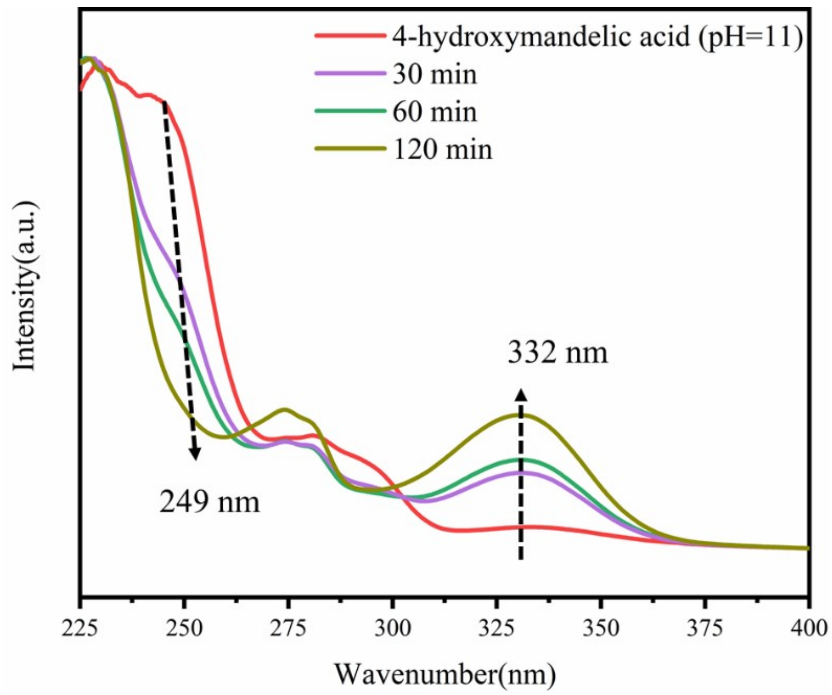

Fig. S8 The UV-vis spectra of 4-hydroxymandelic acid under irradiation.
